# Acute changes in myocardial tissue characteristics during hospitalization in patients with COVID-19

**DOI:** 10.3389/fcvm.2023.1097974

**Published:** 2023-02-16

**Authors:** Mayooran Shanmuganathan, Rafail A. Kotronias, Matthew K. Burrage, Yujun Ng, Abhirup Banerjee, Cheng Xie, Alison Fletcher, Peter Manley, Alessandra Borlotti, Maria Emfietzoglou, Alexander J. Mentzer, Federico Marin, Betty Raman, Oxford Acute Myocardial Infarction (OxAMI) investigators, Elizabeth M. Tunnicliffe, Stefan Neubauer, Stefan K. Piechnik, Keith M. Channon, Vanessa M. Ferreira

**Affiliations:** ^1^Acute Vascular Imaging Center (AVIC), University of Oxford, John Radcliffe Hospital, Oxford, United Kingdom; ^2^Oxford Center for Clinical Magnetic Resonance Research (OCMR), John Radcliffe Hospital, Oxford, United Kingdom; ^3^Oxford University Hospitals NHS Foundation Trust, John Radcliffe Hospital, Oxford, United Kingdom; ^4^Faculty of Medicine, University of Queensland, Brisbane, QLD, Australia; ^5^Institute of Biomedical Engineering, Department of Engineering Science, University of Oxford, Oxford, United Kingdom; ^6^Wellcome Center for Human Genetics, University of Oxford, Oxford, United Kingdom

**Keywords:** SARS-COV2, COVID-19, cardiovascular magnetic resonance imaging, T1-mapping, myocardial edema, T2-weighted images

## Abstract

**Background:**

Patients with a history of COVID-19 infection are reported to have cardiac abnormalities on cardiovascular magnetic resonance (CMR) during convalescence. However, it is unclear whether these abnormalities were present during the acute COVID-19 illness and how they may evolve over time.

**Methods:**

We prospectively recruited unvaccinated patients hospitalized with acute COVID-19 (*n* = 23), and compared them with matched outpatient controls without COVID-19 (*n* = 19) between May 2020 and May 2021. Only those without a past history of cardiac disease were recruited. We performed in-hospital CMR at a median of 3 days (IQR 1–7 days) after admission, and assessed cardiac function, edema and necrosis/fibrosis, using left and right ventricular ejection fraction (LVEF, RVEF), T1-mapping, T2 signal intensity ratio (T2SI), late gadolinium enhancement (LGE) and extracellular volume (ECV). Acute COVID-19 patients were invited for follow-up CMR and blood tests at 6 months.

**Results:**

The two cohorts were well matched in baseline clinical characteristics. Both had normal LVEF (62 ± 7 vs. 65 ± 6%), RVEF (60 ± 6 vs. 58 ± 6%), ECV (31 ± 3 vs. 31 ± 4%), and similar frequency of LGE abnormalities (16 vs. 14%; all *p* > 0.05). However, measures of acute myocardial edema (T1 and T2SI) were significantly higher in patients with acute COVID-19 when compared to controls (T1 = 1,217 ± 41 ms vs. 1,183 ± 22 ms; *p* = 0.002; T2SI = 1.48 ± 0.36 vs. 1.13 ± 0.09; *p* < 0.001). All COVID-19 patients who returned for follow up (*n* = 12) at 6 months had normal biventricular function, T1 and T2SI.

**Conclusion:**

Unvaccinated patients hospitalized for acute COVID-19 demonstrated CMR imaging evidence of acute myocardial edema, which normalized at 6 months, while biventricular function and scar burden were similar when compared to controls. Acute COVID-19 appears to induce acute myocardial edema in some patients, which resolves in convalescence, without significant impact on biventricular structure and function in the acute and short-term. Further studies with larger numbers are needed to confirm these findings.

## Introduction

Early studies of COVID-19 survivors at 3–6 months post-infection found a high prevalence (26–78%) of cardiac abnormalities when evaluated using cardiovascular magnetic resonance imaging (CMR), mainly reporting high myocardial T1 and T2 signals (1–3). However, a case–control study found no excess in CMR abnormalities at 6 months after mild COVID-19 (4). These discrepancies may be multi-factorial, including heterogeneity in the severity of COVID-19 and prevalence of past cardiac disease, scan timing, and protocols (T1 and T2 assessments did not offer full heart coverage in most published studies). Additionally, there was also a lack of systemic testing for biochemical evidence of acute myocardial injury (Troponin rise) at the time of acute illness and lack of control groups (2). Importantly, it is unclear if the myocardial T1 and T2 abnormalities found during convalescence were present during the acute COVID-19 illness. Elevated T1 and T2 indicate elevated myocardial water content, and has been observed in patients with other acute non-cardiac systemic illnesses (5); these may not necessarily indicate histopathologic myocarditis, but may reflect acute myocardial edema. Thus, in this prospective study, we investigated the cardiac findings during the acute and recovering phases of COVID-19 using CMR, in patients without a history of cardiac disease, and compared them to controls without COVID-19 but with matching cardiovascular risk factors.

## Materials and methods

Two cohorts of participants (total *n* = 42) were prospectively recruited near the start of the COVID-19 pandemic, between May 2020 and May 2021: (1) patients hospitalized for acute COVID-19 (*n* = 23) and (2) outpatient controls without COVID-19 but with cardiovascular risk factors and no cardiac symptoms (*n* = 19). Ethical approval was obtained (REC:10/H0408/24); all participants gave written informed consent. The acute COVID-19 cohort included patients admitted with hypoxia requiring oxygen and/or steroid therapy. COVID-19-negative controls were prospectively recruited from the community to match the clinical characteristics (age, gender, and cardiovascular risk factors) in the acute COVID-19 cohort. Exclusion criteria included documented history of pre-existing cardiac disease or cardiac symptoms (verified by electronic patient records), previous COVID-19 and/or vaccination (verified on history and with dedicated antibody testing), and hemodynamic or respiratory instability (i.e. escalating oxygen support) at the point of scanning. Cardiac Troponin levels (cTnI) were measured before the CMR scan. Normal Troponin level at our institution is <34 ng/L. Patients with a clinical diagnosis of acute myocardial infarction (MI) or a clinical indication for CMR were excluded; this was done to investigate the incidental cardiac findings on CMR during acute COVID-19. COVID-19 severity was graded on the World Health Organization (WHO) four-point scale on chest imaging (6). All patients were recruited after they became clinically and hemodynamically stable, and underwent blood sampling and CMR, per our prospective research protocol. Safety precautions, including the wearing of full personal protection equipment (PPE) by researchers, were implemented in line with hospital-wide guidance at the time of the study.

The CMR (3-Tesla) methods included whole-heart slice-matched cine, native T1-mapping (ShMOLLI), bright-blood T2-weighted imaging (T2 signal intensity ratio of myocardial vs. skeletal muscle; T2SI), late gadolinium enhancement (LGE) imaging and extracellular volume (ECV) quantification (7–10). Acute COVID-19 patients were invited for a 6-month follow-up CMR and blood tests. Image analysis was performed on CVI_42_ (Circle Cardiovascular Imaging Inc., Canada) by three experts (MS, MB, and AB). Abnormally high myocardial T1 and T2SI were defined as T1 > 1,244 ms (>2SD above mean in healthy volunteers; 1,184 ± 30 ms) and T2SI > 1.4 (>2SD above mean in healthy volunteers and rounded up; 1.2 ± 0.1), respectively.

Statistical analysis was performed on SPSS v25 (SPSS Inc., United States). Categorical values are presented as frequencies and percentages. Continuous values are presented as mean ± SD or median (IQR), where applicable. Between-group comparisons were performed using independent and paired *t*-tests, Wilcoxon signed-rank test, Kruskal-Wallis test, Fisher’s exact test or McNemar’s test, as appropriate.

## Results

Acute COVID-19 patients and controls had similar baseline characteristics, although there was a trend toward higher proportion of smokers in the acute COVID-19 cohort (11 vs. 40%, *p* = 0.075; Table 1). 65% of acute COVID-19 patients had ≥ WHO grade 2 disease severity on chest imaging; 22% had received non-invasive ventilation; and 13% had a diagnosis of pulmonary embolism prior to the CMR scan. CTnI was elevated in 8 (35%) patients with acute COVID-19, which were considered by the clinical care team to be mild and part of their acute illness (median rise of 2.4-fold above the normal threshold), and thus these patients were not on treatment for acute coronary syndrome or myocarditis. None of the patients had any documented arrhythmia prior to recruitment.

**Table 1:** Baseline characteristics and blood test results.

	Control (*n* = 19)	Acute COVID-19 (*n* = 23)	*p*-value
Age (years)	57 ± 12	58 ± 14	0.838
Gender (Male %)	63	69	0.748
Ethnicity (White Caucasian %)	74	70	1
Past history of cardiac disease	0	0	1
Diabetes (%)	0	9	0.492
Hypertension (%)	16	35	0.291
Smoker (%)	11	40	0.075
Temperature	37.13 ± 0.5	37.96 ± 0.71^*^	<0.001
Oxygen saturation (%)	97.37 ± 1.8	90.13 ± 4.61^*^	<0.001
Systolic blood pressure (mmHg)	136 ± 16	103 ± 23^*^	<0.001
Heart rate (bpm)	67 ± 7	98 ± 17^*^	<0.001
White blood cell count (10^9^/L)	5.63 ± 1.9	7.1 ± 3.9^*^	0.143
Lymphocyte count (10^9^/L)	1.59 (1.42–1.94)	0.69 (0.48–1.00)^*^	<0.001
Hematocrit	0.43 ± 0.03	0.40 ± 0.04	0.031
D-dimer (ng/ml)	227 (167–317)	1,609 (769–2,723)^*^	<0.001
C-reactive protein (mg/L)	1 (0.60–1.5)	120 (60–160)^*^	<0.001
Troponin-I (ng/ml)	1.99 (1.99–3)	9 (5–62)^*^	<0.001
Elevated Troponin (>34 ng/ml; %)	0	35	0.005
NT-pro-BNP (pg/ml; normal <400 pg/ml)	53 (25–77)	217 (70–365)^*^	0.001
COVID-19 severity score ≥ 2 on CT	N/A	65	N/A
Pulmonary embolism diagnosed (%)	N/A	13	
Treatment with steroids (%)	N/A	57	
Treatment with Remdesevir (%)	N/A	48	
Treatment with antibiotics (%)	N/A	74	
Non-invasive ventilation (%)	N/A	22	

Baseline demographics, vital signs, blood test results (^*^ worst values during hospitalization for COVID-19 patients), and treatment administered and CMR findings. Categorical values are presented as frequencies in percentage (%). Continuous values are presented as mean ± SD or median (IQR) where applicable. CTnI levels reported as < 2 ng/ml in the laboratory assay are presented as 1.99 ng/ml. NT-pro-BNP, N-Terminal prohormone of brain natriuretic peptide; CT, computed tomography.

Median time between admission for COVID-19 infection and CMR was 3 days (1–7). On CMR imaging, controls and acute COVID-19 patients had similar left and right ventricular ejection fraction (LVEF 62 ± 7 vs. 65 ± 6%; *p* = 0.103, RVEF 60 ± 6 vs. 58 ± 6%; *p* = 0.373). They also had similar ECV (31 ± 3 vs. 21 ± 4%; *p* = 0.0804) and frequency of LGE abnormalities (16 vs. 14%; *p* = 0.981). Non-ischemic LGE, suggestive of myocarditis, was observed in two participants each from the control (11%) and acute COVID-19 (9%) cohorts. Myocardial infarction was present in one each from the two cohorts (Table 2).

**Table 2:** CMR and blood test findings in controls, acute COVID-19 patients, and at follow up.

	Controls (*n* = 19)	Acute COVID-19 (*n* = 23)	*p*-value *(vs controls)*	Follow up COVID-19 (*n* = 12)	*p*-value *(vs controls)*	*p*-value *(vs acute; paired analysis)*
Time of CMR scan after admission (days)	N/A	3 (1–7)	N/A	166 (116–184)	N/A	N/A
Heart rate during scan (bpm)	67 ± 7	72 ± 15	0.077	65 ± 11	0.571	0.199
LVEDV indexed to BSA (ml/m^2^)	74 ± 13	67 ± 15	0.103	75 ± 13	0.920	0.761
Left ventricular ejection fraction (%)	62 ± 7	65 ± 6	0.272	65 ± 7	0.397	0.615
RVEDV indexed to BSA (ml/m^2^)	77 ± 15	69 ± 15	0.118	72 ± 14	0.356	0.975
Right ventricular ejection fraction (%)	60 ± 6	58 ± 6	0.373	61 ± 6	0.490	0.085
Global LV myocardial T1 (ms)	1,183 ± 22	1,217 ± 41	**0.002**	1,180 ± 36	0.435	0.177
Global myocardial T2 Signal Intensity ratio (T2SI)	1.13 ± 0.09	1.47 ± 0.36	**<0.001**	1.24 ± 0.09	**0.006**	0.127
Global myocardial extracellular volume (ECV) (%)	31 ± 3	31 ± 4	0.804	29 ± 3	0.073	0.234
Presence of pathological LGE^§^, *n* (%)	3 (16)	3 (14)	1	0 (0)	N/A	N/A
Presence of ischemic LGE, *n* (%)	1 (5)	1 (5)	0.981	0 (0)	N/A	N/A
Presence of non-ischemic LGE, *n* (%)	2 (11)	2 (9)		0 (0)	N/A	N/A
C-reactive protein (mg/L)	1 (0.60–1.5)	120 (60–160)	**<0.001**	1.4 (1–2.2)	0.104	**0.005**
Troponin-I (ng/ml; normal <34 ng/ml)	1.99 (1.99–3)	9 (5–62)	**<0.001**	4.1 (1.99–7.3)	0.085	**0.004**
NT-pro-BNP (pg/ml; normal <400 pg/ml)	53 (25–77)	217 (70–365)	**0.005**	83 (36–230)	0.346	**0.033**

Baseline CMR findings. Categorical values are presented as frequencies in percentage (%). Continuous values are presented as mean ± SD or median (IQR) where applicable. LVEDV, left ventricular end diastolic volume; BSA, body surface area; ECV, extracellular volume. Myocardial T2 signal intensity ratio (T2SI) was compared to skeletal muscle. LGE, late gadolinium enhancement. Other abbreviations as per Table 1

§ Presence of LGE in the ventricular insertion points was not considered pathological. The patterns of non-ischemic LGE seen in each group: two control subjects had sub-epicardial LGE and two COVID-19 patients had mid-wall fibrosis. *p* values less than <0.05 are in bold. N/A - Not applicable.

**Figure 1:**
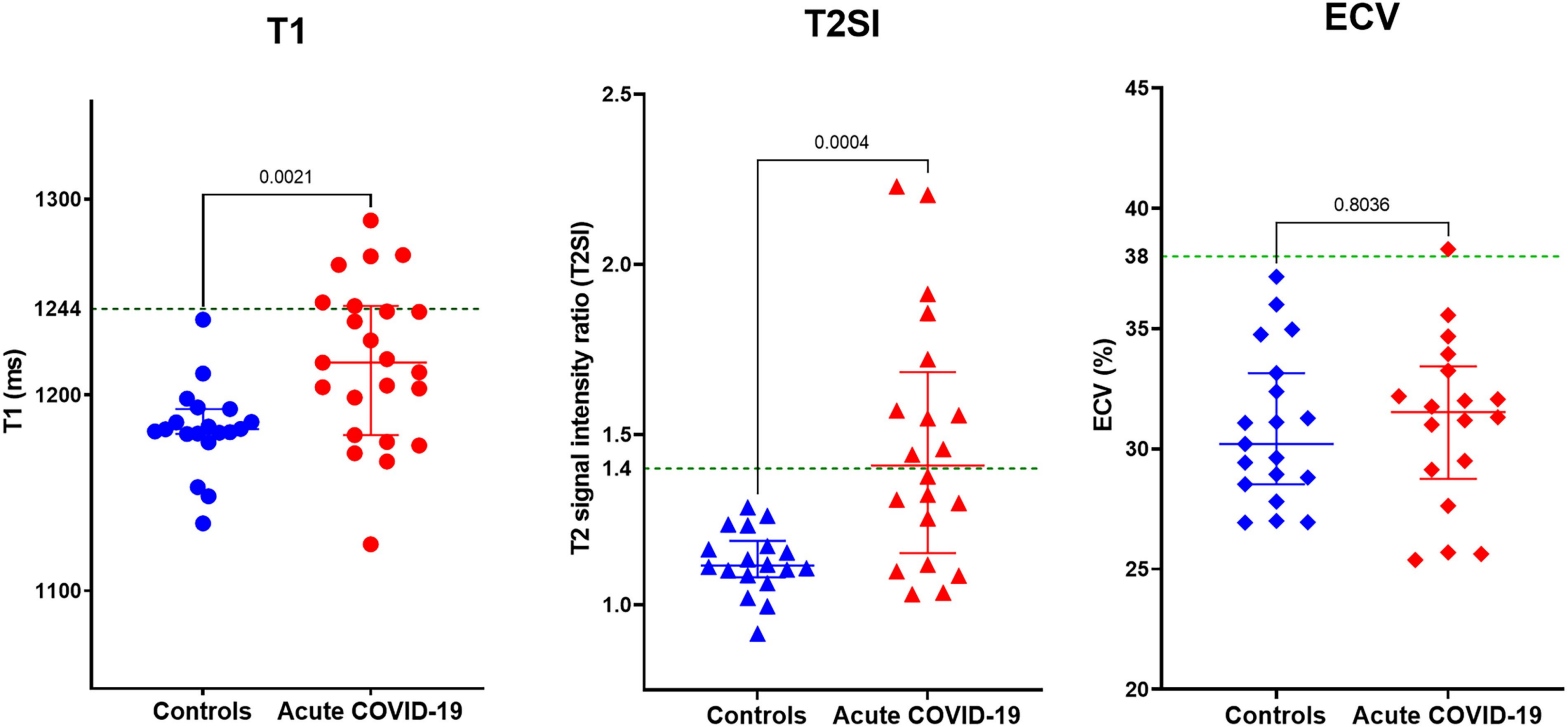
Myocardial tissue characteristics on cardiovascular magnetic resonance (CMR) during acute COVID-19 in hospitalized patients compared to controls without COVID-19 from the community matched for cardiovascular risk factors. Comparison of global myocardial T1, T2 signal intensity (T2SI) ratio, and extracellular volume (ECV) fraction between outpatient controls without COVID-19 and patients with acute COVID-19 during their hospitalization. Green dotted line in the graphs denotes the upper limit of normal ranges for T1, T2SI ratio, and ECV in the 3 T MR scanner used for this study.

Compared to controls, patients with acute COVID-19 had significantly higher myocardial T1 (1,183 ± 22 vs. 1,217 ± 41 ms; *p* = 0.002) and T2SI (1.13 ± 0.09 vs. 1.47 ± 0.36; *p* < 0.001; Figure 1). Whilst all control subjects had normal T1 and T2SI, abnormally high T1 and T2SI were present in 26 and 50% of acute-COVID patients, respectively. Those with abnormally high T1 had significantly higher LVEF compared to patients with normal T1 (68 ± 8 vs. 62 ± 5%; *p* = 0.04), while there was no significant difference in the LVEF between patients with abnormally high and normal T2SI (65 ± 7 vs. 63 ± 6%; *p* = 0.519). There were no significant differences in the T1, T2SI, or ECV between patients treated with and without corticosteroids [1,220 ± 42 vs. 1,213 ± 41 ms (*p* = 0.703), 1.43 ± 0.34 vs. 1.53 ± 0.40 (*p* = 0.543), and 32 ± 4 vs. 30 ± 4% (*p* = 0.303), respectively].

**Figure 2:**
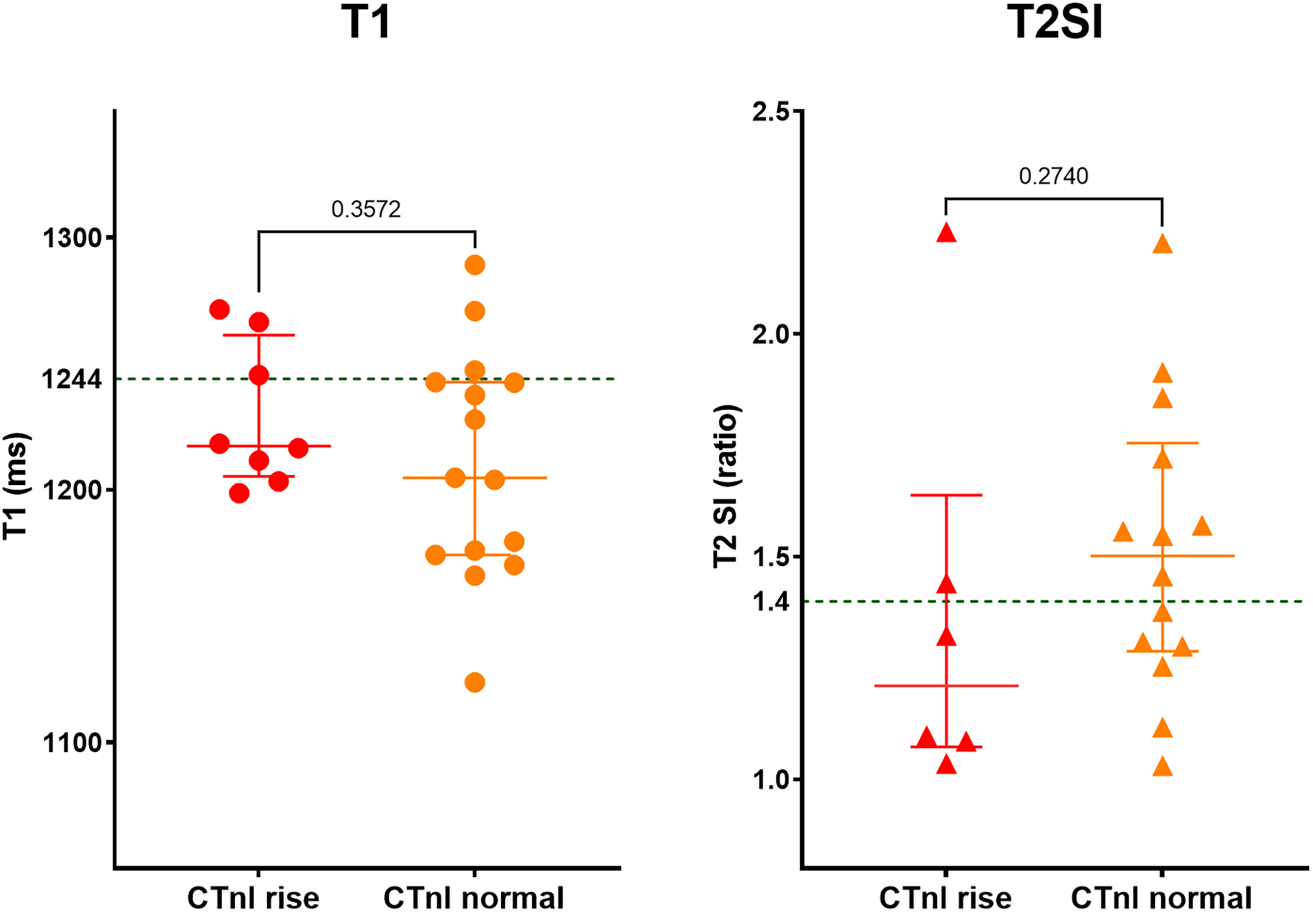
Myocardial tissue characteristics on CMR during acute COVID-19 in hospitalized patients with and without mild cardiac Troponin (CTnI) rise. Out of 23 patients, eight had mildly elevated CTnI levels (on average 2.4-fold above normal range). Green dotted line in the graphs denotes the upper limit of normal range for T1 and T2SI ratio in the 3 T MR scanner used for this study.

The acute COVID-19 patients with mildly elevated CTnI had similar CMR findings compared to acute COVID-19 patients with normal CTnI levels; the T1 was 1,217 ± 28 vs. 1,205 ± 45 ms (*p* = 0.357) and the T2SI was 1.51 ± 0.33 vs. 1.37 ± 0.45 (*p* = 0.274), respectively (Figure 2). Pathological LGE was seen in 2/8 patients with raised CTnI and 1/15 patients with normal CTnI levels, leading to further cardiology referrals, and coronary revascularization in one patient. The CMR findings did not change the clinical management of other patients.

**Figure 3:**
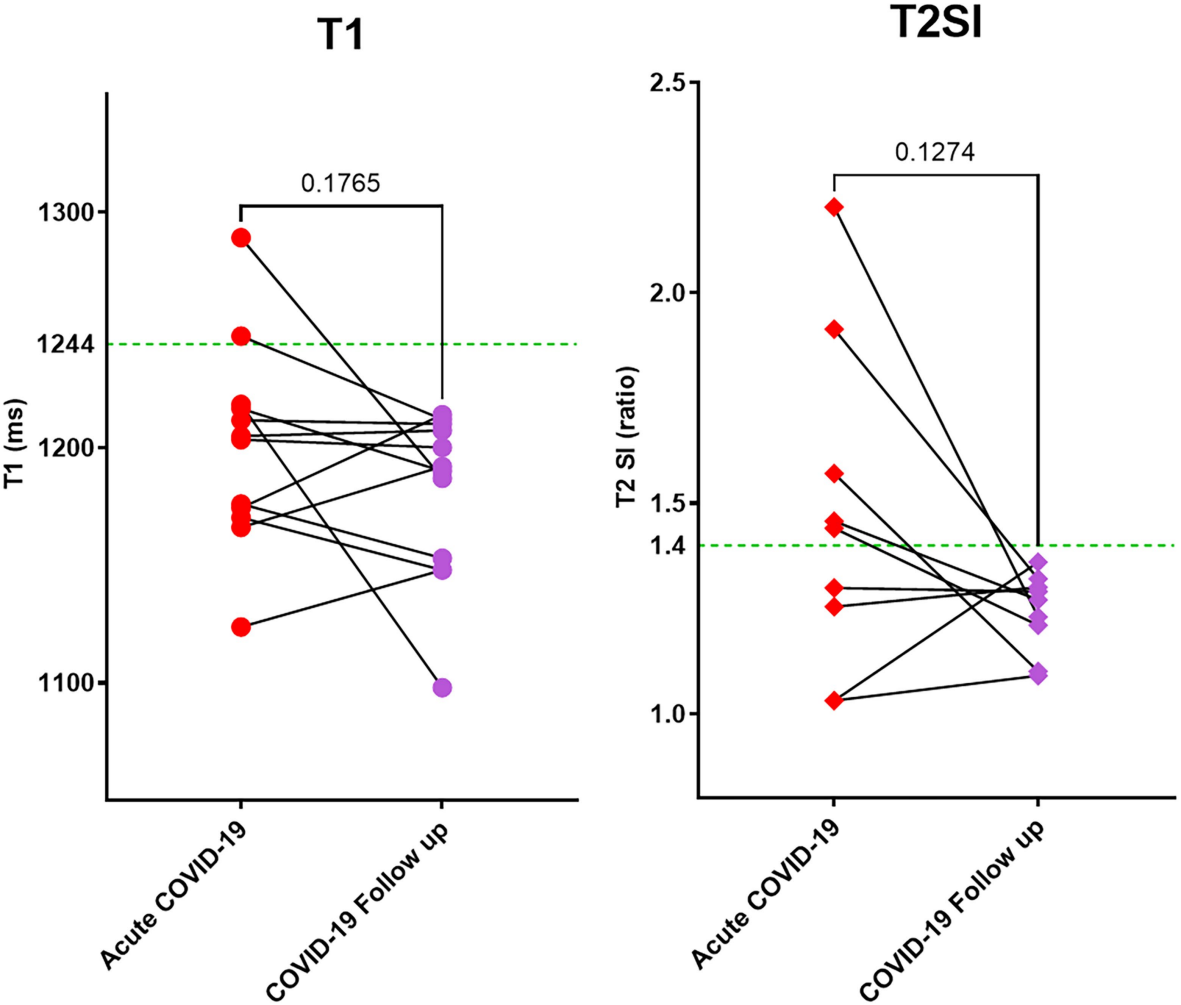
Myocardial tissue characteristics as measured by CMR T1 mapping and T2 weighted imaging. During acute COVID-19 illness and at follow up (at 6 months). Out of 23 patients scanned during acute COVID-19, 12 returned for follow up during the pandemic. All patients had normal T1 and T2SI ratio at follow up. Green dotted line in the graphs denote the upper limit of normal range for T1 and T2SI ratio in the 3 T MR scanner used for this study.

Twelve acute COVID-19 patients returned for follow-up at around 6 months (median 166, IQR 116–184 days) after the acute CMR scan. At follow up, patients had normal inflammatory [CRP 1.4 (1–2.2) mg/L] and cardiac biomarkers [Troponin-I 4.1 (1.99–7.3 ng/L); NT-proBNP 83 (36–230) pg./ml; all *p* < 0.05 when compared to blood tests during the hospitalization; Table 2]. All patients demonstrated normal myocardial T1 (1,180 ± 36 ms) and T2SI (1.24 ± 0.09) on the 6-month scan (Table 2; Figure 3). There were no new LGE abnormalities at follow up.

## Discussion

**Figure 4:**
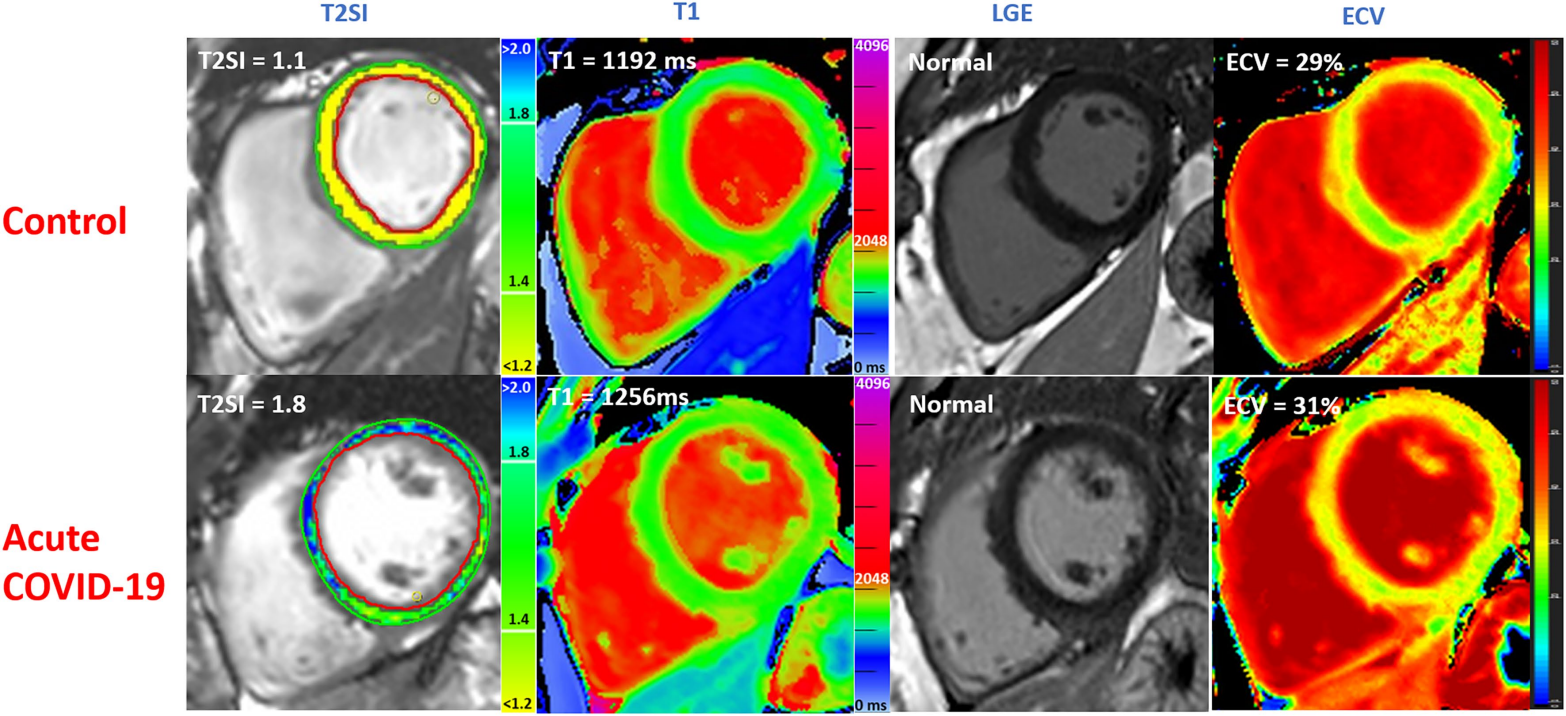
Multiparametric CMR imaging in 3 T MRI scanner comparing acute COVID-19 patients during their hospitalization with matched controls without COVID-19 from the community. T2SI = T2 signal intensity ratio (myocardium: skeletal muscle). LGE, late gadolinium enhancement; ECV, extra cellular volume fraction. Normal values: T1 ≤ 1,244 ms, T2 ≤ 1.4. All values correspond to single slice have been shown.

For the first time, we describe the acute cardiac findings using CMR in hospitalized patients with acute COVID-19, and compared them to matched controls and performed follow-up scans at 6 months. We found that acute COVID-19 was associated with significant myocardial edema in some patients, as demonstrated by significantly elevated myocardial T1 and T2 signals compared to controls (Figure 4). Otherwise, both cohorts had normal biventricular systolic function, similar ECV fraction and frequency of LGE findings. Given the similar ECV fraction between acute COVID-19 patients and controls, we propose that the mechanism of raised T1 and T2 signals in acute COVID-19 may, in part, be attributed to the presence of intracellular edema. Findings from the follow up CMR scans suggest that the acute myocardial edema tend to normalize over time during the convalescent phase, with preserved biventricular function.

SARS-CoV2 has been reported to induce acute myocardial injury and heart failure, similar to other cardiotropic viruses (11, 12). Earlier studies of COVID-19 survivors in the community reported significantly elevated myocardial T1 and T2 signals on CMR in convalescence; but the pathophysiologic origins of these imaging findings were unclear, especially in the absence of evidence for acute myocardial injury (troponin rise and fall) during the acute illness and the inclusion of patients with previous history of cardiac disease (2, 13). T1 and T2 signals can be prolonged due to increased myocardial water content, regardless of injury to the cardiomyocytes or histopathological myocarditis. Such a change in water content can take place in either or both intracellular or extravascular (including coronary arteries) compartments and be attributed to physiological changes during stress (14, 15). Therefore, it is possible that similar alterations may occur during a systemic inflammatory condition such as acute COVID-19, leading to elevation in T1 and T2 values, which then regress to normal during convalescence.

To our knowledge, the only study to-date to describe findings in acutely ill COVID-19 patients was performed by Chen et al. (16): it was retrospective study of a young cohort (median age 23 years) of well COVID-19 patients (*n* = 25) with suspected myocardial injury [Troponin rise (*n* = 8) or ECG changes or cardiac symptoms] at ~6 days after symptoms onset, and who had a clinical indication for CMR. They observed higher T1, T2, and ECV fraction when compared to healthy controls. In contrast, given the association of older age with negative clinical outcomes (17), our prospective study recruited older (mean age 56 years) and sicker acute COVID-19 patients without a clinical indication for CMR. Furthermore, to reduce the effects of confounding factors, we excluded patients with a history of cardiac disease, and included a control group with similar cardiovascular risk factors, measured cardiac troponin levels acutely, investigated their associations with the imaging findings, and performed follow-up CMR imaging.

We observed that myocardial T1 and T2SI abnormalities in acute COVID-19 patients with mildly elevated cTnI levels were not different from acute COVID-19 patients who had normal cTnI levels. This is similar to the observation made by Chen et al. (16). This suggests that myocardial edema in COVID-19 can occur independently of troponin rise. Moreover, we found no differences in the frequency of LGE abnormalities between controls and acute-COVID patients (who were recruited on the basis of no documented history of cardiac disease). This suggests that the LGE abnormalities described in recent studies of patients during the recovering phase of COVID-19 could have been pre-existing, pre-dating the acute COVID-19 illness; thus longitudinal studies of healthy subjects who had CMR scans performed pre-pandemic are needed (2). Furthermore, while patients in our study with mild rise in cTnI exhibited similar CMR findings to those with normal CTnI, in clinical practice, the magnitude of cTnI and clinical context, especially the presence of past history of cardiovascular disease, need to be carefully evaluated. Moreover, we did not detect any arrhythmias, a potential cause of Troponin rise, in our cohort during the hospital admission; but previous reports have documented arrhythmias during acute COVID-19 (18, 19).

The findings from our follow-up of acute COVID-19 patients on their 6-month CMR suggest that the myocardial tissue abnormalities (T1, T2) improved (and normalized) in convalescence, in keeping with previous reports (20, 21). The preservation of global biventricular systolic function during both the acute and recovery phases of COVID-19 in our study supports evidence from other reports that T1 and T2 abnormalities were not correlated with biochemical or imaging evidence of heart failure at the time of assessment during the recovery phase (1, 2, 22). Furthermore, the presence of higher LVEF in patients with abnormally high myocardial T1 raises the possibility that acute COVID-19 leads to a hyperdynamic state resulting in increased myocardial blood volume (plasma and red blood cells), which may have been detected as higher myocardial T1 (15). Such a hyperdynamic state would be expected to return to baseline after the resolution of the acute illness resulting in normalization of T1.

## Limitations

Our study has some limitations. This is a small study that was confined to hospitalized acute COVID-19 patients and conducted before the launch of mass vaccination programs; thus, the findings may not be applicable to those managed in the community or those who have had the COVID-19 vaccine. Due to pandemic restrictions and patient preferences, we were unable to complete follow-up assessments in all COVID-19 patients, which may have under-powered some of the results; such as the lack of statistical significance in the differences of T1 and T2SI between the acute and follow-up time points. The thinness of the RV free wall renders it almost impossible to accurately quantify its T1 and T2 characteristics; thus, important pathophysiological changes in the RV myocardium secondary to acute COVID-19, which can increase the RV afterload, cannot be ascertained here (23). The influences of the differences in pathophysiology (e.g., hypoxia) and treatment (e.g., oxygen therapy and steroids) in acute COVID-19 on myocardial T1 and T2 signals were not explored in this study. On average, we scanned our patients at ~3 days into their hospitalization with acute COVID-19; it is unclear if the infection may lead to different or more myocardial abnormalities at a later time point during the acute illness. Whilst 65% of our patients had moderate or severe grade of COVID-19 illness on chest imaging (Table 2), we recruited relatively stable patients and thus our findings may not be generalizable to a sicker cohort. It is not yet clear if myocardial edema associated with SARS-COV2 illness is unique or whether similar changes happen with other viral illnesses. Thus, our study’s findings should be viewed as hypothesis-generating, and may inform future study designs with a larger sample of patients with the ability to compare between vaccinated and unvaccinated patients, as well other cohorts of patients with different viral illnesses.

## Conclusion

In this prospectively conducted study of participants with no history of cardiac disease or COVID-19 vaccination, patients hospitalized for acute COVID-19 demonstrated acute myocardial edema on CMR when compared to controls with similar cardiovascular risk factors, which normalized at 6 months. Biventricular systolic function was normal and scar burden was low and similar between the two groups. Acute COVID-19 appears to induce acute myocardial edema in some patients, which resolves in convalescence, without significant impact on biventricular structure and function in the acute and short-term. Further studies with larger numbers are needed to confirm these findings.

## Data availability statement

The original contributions presented in the study are included in the article/supplementary material, further inquiries can be directed to the corresponding author.

## Ethics statement

The studies involving human participants were reviewed and approved by NHS Health Research Authority, NRES Committee South Central—Oxford C. REC reference number: 10/H0408/24. The patients/participants provided their written informed consent to participate in this study.

## Author contributions

MS, VF, and KC conceived and designed the study. MS, YN, FM, and RK recruited study participants and performed blood sampling and analysis. AM helped to design the study and blood analysis. ME performed blood analysis and data collection and curation. MS, MB, VD, CX, and ABo performed image analysis. BR, SN, and ET provided scientific advice. VF, KC, and SP provided supervision of the project. MS and ABa performed statistical analysis. MS and VF prepared the manuscript. All authors contributed to the article and approved the submitted version.

## Funding

This work was supported by the British Heart Foundation (CH/16/1/32013), BHF Center of Research Excellence, Oxford (RG/13/1/30181 and RE/18/3/34214), and National Institute for Health Research (NIHR) Oxford Biomedical Research Center. MS was funded by the Alison Brading Memorial Graduate Scholarship in Medical Science, Lady Margaret Hall, University of Oxford. MB acknowledges support from a British Heart Foundation Clinical Research Training Fellowship (FS/19/65/34692). VF and SP acknowledge the BHF, BHF Oxford CRE, and NIHR Oxford BRC for support. ABa is a Royal Society University Research Fellow and is supported by the Royal Society (grant no. URF\R1\221314).

## Conflict of interest

SP has patent authorship rights for U.S. patent 9285446 B2 [systems and methods for Shortened Look Locker Inversion Recovery (Sh-MOLLI) cardiac gated mapping of T1], granted March 15, 2016; IPs are owned and managed by Oxford University Innovations.

The remaining authors declare that the research was conducted in the absence of any commercial or financial relationships that could be construed as a potential conflict of interest.

## Publisher’s note

All claims expressed in this article are solely those of the authors and do not necessarily represent those of their affiliated organizations, or those of the publisher, the editors and the reviewers. Any product that may be evaluated in this article, or claim that may be made by its manufacturer, is not guaranteed or endorsed by the publisher.
